# Collecting real-time infant feeding and support experience: co-participatory pilot study of mobile health methodology

**DOI:** 10.1186/s13006-025-00707-7

**Published:** 2025-04-03

**Authors:** Abigail E. Page, Emily H. Emmott, Rebecca Sear, Nilushka Perera, Matthew Black, Jake Elgood-Field, Sarah Myers

**Affiliations:** 1 Centre for Culture and Evolution, Brunel University London, London, UK; 2 London School of Hygiene and Tropical Medicine, Population Health, London, UK; 3 Department of Anthropology, University College London, London, UK; 4 Best Beginnings, London, UK; 5 Department of Health and Social Care, London, UK; 6 BirthRites Lise Meitner Research Group, Max Planck Institute for Evolutionary Anthropology, Leipzig, Germany

**Keywords:** Human-centred design, Infant feeding, Social support, MHealth, Co-production

## Abstract

**Background:**

Breastfeeding rates in the UK have remained stubbornly low despite long-term intervention efforts. Social support is a key, theoretically grounded intervention method, yet social support has been inconsistently related to improved breastfeeding. Understanding of the dynamics between infant feeding and social support is currently limited by retrospective collection of quantitative data, which prohibits causal inferences, and by unrepresentative sampling of mothers. In this paper, we present a case-study presenting the development of a data collection methodology designed to address these challenges.

**Methods:**

In April–May 2022 we co-produced and piloted a mobile health (mHealth) data collection methodology linked to a pre-existing pregnancy and parenting app in the UK (Baby Buddy), prioritising real-time daily data collection about women's postnatal experiences. To explore the potential of mHealth in-app surveys, here we report the iterative design process and the results from a mixed-method (explorative data analysis of usage data and content analysis of interview data) four-week pilot.

**Results:**

Participants (*n* = 14) appreciated the feature’s simplicity and its easy integration into their daily routines, particularly valuing the reflective aspect akin to journaling. As a result, participants used the feature regularly and looked forward to doing so. We find no evidence that key sociodemographic metrics were associated with women’s enjoyment or engagement. Based on participant feedback, important next steps are to design in-feature feedback and tracking systems to help maintain motivation.

**Conclusions:**

Reflecting on future opportunities, this case-study underscores that mHealth in-app surveys may be an effective way to collect prospective real-time data on complex infant feeding behaviours and experiences during the postnatal period, with important implications for public health and social science research.

**Supplementary Information:**

The online version contains supplementary material available at 10.1186/s13006-025-00707-7.

## Introduction

Breastfeeding is a key intervention target to improve mother-infant-outcomes. Worldwide, many mothers do not breastfeed for the WHO recommended durations [1]. The UK has the lowest proportion globally meeting this target: fewer than 1% of infants are exclusively breastfed for six months [2]. Breastfeeding rates also show socioeconomic gradients, with socioeconomically disadvantaged women in high-income countries (HIC) breastfeeding at lower rates than advantaged women [2]. Given breastfeeding is potentially linked to inflammation in infants and infant weight, these differences in breastfeeding rates may contribute to socioeconomic gradients in infant outcomes [3]. It has been estimated that, if 45% of UK mothers exclusively breastfed for four months, a financial saving of £31 million [4] would be made via mitigating physical and mental health costs for both mothers and children [2, 5]. Maternal mental health is closely intertwined with infant feeding experiences [6, 7] and the pressure to breastfeed as ‘best’ or ‘moral’ can negatively impact mothers when women face the reality of infant feeding [8]. Thus, being able to support women’s infant feeding goals is important for improving maternal and infant outcomes and the postnatal experience.

Theoretically, social support—in multiple forms—during the vulnerable postnatal period is profoundly important and acts via multiple pathways to impact breastfeeding [9]; the performance of practical support by others can increase maternal energetic resources [9–12], while receipt of advice (informational support) and empathy (emotional support), can increase the mother's ability to cope [13–17]. It is puzzling then, that social support has not been consistently associated with beneficial outcomes [17]. For instance, randomised control trials of social support in the UK have had limited or no sustained impact on breastfeeding duration [18–20]. This suggests that our understanding of social support in the postnatal period is not currently sufficient. Overall, it seems we do not yet have a clear understanding of the factors which influence breastfeeding. Despite increasing breastfeeding rates being a focal point of health policy in England for decades [14, 21], there was only minimal improvement in initiation rates between 2010 and 2022 (73.7% initiated in 2010–2011 compared to 74% in 2022). A similar picture is apparent for breastfeeding rates at 6 – 8 weeks postpartum [5, 22]. We have argued elsewhere that a focus on social support is warranted, for theoretical reasons, but that a more granular approach to social support is required, taking into account the varying pathways by which different types of support act [9, 13]. Two further key barriers to understanding social support in the postnatal period are 1) retrospective and cross-sectional quantitative study designs and 2) unrepresentative samples. Here, we report on progress towards developing a data collection methodology to overcome these issues.

### Retrospective quantitative study designs

While qualitative studies have collected extensive and rich data on the lived experience of infant feeding, sample sizes are relatively small, limiting generalisations [23]. Quantitative studies have larger samples yet are often retrospective due to difficulties in data collection during the early postpartum period. Retrospective data are subject to recall bias, which may be heightened by the intensity of the first weeks following childbirth. Many problems relating to infant feeding are most acute early on, meaning crucial information regarding the timing and sequence of events may be obscured by reporting error retrospectively. New mothers may also forget key early events in the light of later ones when asked to summarise events over longer time periods [23]. Previous experiences can be reported differently, or not at all, due to later experiences as people update their narratives in hindsight. Prospectively collected data are therefore necessary if we are to reliably understand cause and effect, which will ultimately determine intervention targets [24]. To rectify this knowledge gap, we need in-depth prospective data on social support from the start of the postnatal period [24].

### Unrepresentative samples

A priority research area in the UK, and other high-income countries, is exploring how social support interplays with socioeconomic inequality to impact child health and development [24]. This is currently limited by studies facing issues recruiting and retaining less affluent and ethnically diverse women [24–26], including our own work [13]. The early postnatal period, and infant feeding in particular, is difficult and often all-consuming. Mothers have little spare time, desire or cognitive capacity to participate in studies which can be labour intensive. This reduces diversity in enrolled participants along social, demographic and economic lines. Furthermore, infant feeding – and particularly breastfeeding – is often associated with ‘good’ motherhood which more privileged sections of society value and promote [27], generating stigma. This lack of diversity undermines our ability to capture the relationship between social support and infant feeding in large sections of society.

Large scale, prospective quantitative surveys of the early postnatal period which capture infant feeding and social support from a diverse sample of women do not exist. To address this gap, we developed and piloted a mobile health (mHealth) methodology, focusing on inclusive, real-time data collection about women's feeding experiences. mHealth involves the use of mobile devices to support medical or public health practice, ranging from data collection, complex interventions or communication devices [28, 29]. Over the last ten years the mHealth space has grown rapidly, with over 58% of patients reporting they downloaded smartphone health apps [30] and a large number of products available on app stores, as well as targeted apps deployed in academic research [28, 31]. While app-based solutions may seem “easy”, to be effective they require extensive iterative research and developmental work with a multidisciplinary team [31]. In particular, the technology must be designed to meet the needs of the stakeholders, with consideration given to how stakeholders may vary in key sociodemographic traits right from the start in the design process [28, 32]. This requires an agile, human-centred design. Human-centred design is an iterative process based on collaborating with users and stakeholders to develop products or services based on their needs, relying on co-participatory methods from the start [32].

### Aims

Here, we document how we used an agile, human-centred design and co-production methodology with a sample of women, including those who are often underrepresented in the literature (less affluent and ethnically diverse women [24–26]). We conducted this pilot study to explore the feasibility of a methodological tool which 1) collects dense data, 2) on a daily basis, 3) during the hectic early postnatal period (for up to 12 weeks), and 4) does not systematically exclude less privileged women by 5) adding value to participants.

To achieve this, we partnered with the charity Best Beginnings (London, UK) to include a data collection survey ‘feature’ to their existing Baby Buddy app. Baby Buddy is a multi-award-winning, UK National Health Service-aligned app to support parents by providing evidence-based information and self-care tools (www.bestbeginnings.org.uk/baby-buddy). It is specifically designed for, and targeted at, mothers from less privileged backgrounds. Working with app producers and users, we co-designed, developed and finalised a minimally viable product (MVP) feature to record women’s postnatal experiences. We then piloted this feature for 4 weeks with 14 participants for beta testing, monitoring usage and fixing bugs. During this period, the lead author met with the participants once a week to incrementally improve functionality, conducting semi-structured interviews to capture their experiences, and modifying the feature in response to their comments as the pilot progressed.

Here, we first detail the app feature development process. We then present an exploratory analysis of quantitative and qualitative data from the interviews and from usage of the feature to review the feasibility of a co-produced mHealth solution for daily prospective data collection in the postnatal period. Given the focus on inclusivity and intersectionality, we review our findings in the light of three goals: 1) the feature should be valuable to women such that they want to use it on a regular basis; 2) participants find it simple and easy to use and 3) the design and development of the feature should not systematically exclude users based on sociodemographic characteristics. Ultimately, we hope this process will result in a robust methodological tool which will address prior limitations in the infant feeding literature, as well as highlighting the process and value of an agile, human-centred design and co-production methodology.

## Method

This project was approved by the London School of Hygiene and Tropical Medicine (LSHTM) Observational Research Ethics Committee A board (reference: 26,171) and was conducted in accordance with the Declaration of Helsinki and relevant national and institutional standards. All participants were provided with information sheets detailing the aims of the research (i.e. feature piloting), risks and benefits and were informed of their right to withdraw from the study at any time without justification. Participants consented both in writing and verbally at the start of the interviews. Participants were compensated for their time with vouchers (of their choice) at the end of the study. Each participant was given £2.50 worth of voucher for each day the app feature was used (£70 max given at the end of the study) and £5 worth of voucher for each interview (£20 max).

### App feature development

It was essential that both stakeholders and ‘users’ had active and leading involvement in the entire research and development process [31, 32]. We achieved this following a purposefully slow and reflective process involving the stakeholders – Best Beginnings and their existing app-development team, the researchers and the participants/users. Given the function of the feature as a data collection tool, it is necessary to ensure that the daily survey is robust and comprehensive. As the feature is integrated within the existing Baby Buddy app it must match the design characteristics of the app, as well as work within its existing environment. Finally, participants need to be able to value using the feature, as well as find it quick and easy to do so, to motivate them to continue using it. To ensure these conditions were achieved, we followed three steps – design strategy, feature design and feature development [31]. First, based on the scientific requirements of the survey the researchers (AEP, EHE, RS, SM) collaborated to develop a design strategy for the feature based on the overarching goals of the project. This involved considering the existing literature on social support and infant feeding which informed what must be included within the feature. To reduce the burden on participants we followed a very simple design and colour scheme, consistent ordering of questions and, in the first instance, only closed questions (multiple choice questions). The text presented within the app has a Flesch Reading Ease score of 70.7 and a Flesch-Kincaid Grade Level of 6.2 which makes it accessible to a 13-year-old in the British school system.

The next step was taking this design strategy to Best Beginnings and the app developers (NP, MB, JE-F) to work with them to develop the feature within the existing Baby Buddy framework. As a team we developed basic sketches of the feature as wireframes (simple, low-fidelity visual layouts that outline the structure and functionality of a user interface) which captured the conditional flow of the daily questions. These were then extensively tested by team members to ensure the wireframes performed as expected and remained easy to use. The design was specifically kept simple at this stage since there had not been any input from participants. Based on the agreed-on wireframes, the app developers from Best Beginnings produced feature v1, which was the minimum viable product (MVP). Feature v1 was then beta tested by team members for one week before the enrolment of participants to ensure bugs were fixed and it was meeting basic expectations. At this point, participants were recruited (detailed below). Given the iterative nature of the human-centred design framework, at each weekly interview participants were specifically asked about bugs, dislikes and suggested improvements. Changes to functionality were made in real-time to improve the feature, and then checked at the next week’s interview to ensure the problem had been solved. Such changes included highlighting that users can ‘scroll down’ when the option list was longer than initially apparent on (particularly smaller) screens, and the inclusion of optional open text boxes for entering further detail for participants who feel like sharing more. We also asked if there were other elements participants would like and benefit from being included in the app to encourage their usage and improve their experience. This final development process is still ongoing as the team integrates more technically complex responses to the findings reported here into version 2.

### Recruitment and sampling

Initial recruitment occurred via convenience sampling among the existing Baby Buddy user base (currently 45,000–55,000 new users annually) from the UK. While convenience-sampling may introduce recruitment bias [15, 33], it is cost and time efficient in a pilot study and the already diverse nature of Baby Buddy’s user base minimised concerns. Pop-up adverts for the study were posted to existing users’ news feeds, which act as the home page when first opening the app. Eligibility criteria were all mothers aged 18 – 45 years who currently have an infant aged 0 – 9 weeks, to ensure that no infant was older than 13 weeks when the trail finished (since the feature is being designed for use in the first 12 weeks following birth). Participants were then taken “off app” to a recruitment survey hosted by formr.org [34] to capture socio-demographic information (age, household income, educational attainment, ethnicity and current feeding mode), allowing us to then perform non-random purposeful sample selection from the initial pool of recruits. After two weeks, 110 participants had signed up for the study. From this sampling frame, we purposively sampled 15 women on the basis of income, ethnicity, educational attainment and current infant feeding status. We oversampled (as compared to the distribution of women who expressed interest) minority ethnic women, women on lower incomes and with lower levels of educational attainment and those non-exclusively breastfeeding. Of the 15 participants selected for the study, one dropped out after the first week and their data were removed from the study.

### Data collection

Quantitative data were collected from the registration survey and from the feature itself in April 2022 to explore if feature usage was structured by ethnicity, income, education, age or infant feeding mode. During the weekly interviews, quantitative data were collected from closed questions to measure participants’ experience. The interviews further gathered qualitative data to better understand the participants needs and experience, adding essential context and explanatory depth to the quantitative results.

#### App-feature data collection

Participants were asked to use the app feature daily for four weeks and here we report an overview of usage patterns. As the focus of this pilot project is user experience, we do not explore the interrelationships between the key indicators the feature is designed to collect (i.e. social support, feeding experience and maternal experience).

#### Weekly interviews

At the end of each week, all participants had a 15-min semi-structured interview with AEP. Participants were asked to rate a number of aspects of their user experience over the previous week: 1) their overall experience of using the feature on a scale of 0 to 10 (with 10 being the ‘best’ experience); 2) if they found the daily survey too long (yes/no); 3) if they found the daily nature of reporting too frequent (yes/no); 4) if they found the repetitive nature of the daily questions acceptable or not (yes/no) (in the last week only), and; 5) how long they spent completing the survey (week 1 only). Participants were then asked to expand on these answers if they would like to, and subsequently what they enjoyed or disliked about the using the feature, any bugs they had experienced, what changes they would make and what would help their motivation to continue using the feature in the future. The interviews were conducted either via the video conferencing platform Zoom or phone, with the audio recorded using a dictaphone and transcribed for a content analysis.

### Analysis

#### Quantitative analysis

The quantitative analysis is broken into four sections: 1) summary statistics about usage, experiences, and how long daily reporting took (self-reported); 2) exploratory data analysis examining if any sociodemographic features associated with the feature usage, experience and how long the survey took; 3) exploratory data analysis examining if there was any relationship between maternal postnatal experience and feature usage; and 4) summary statistics on degree to which responses varied on a daily basis, to assess the need for the daily prospective design.

We ran a series of Poisson models exploring patterning of usage (as measured by number of daily reports, time taken and experience) based on sociodemographic features of participants. Given the small sample size and exploratory nature of this analysis, we do not draw statistical inferences based on the *p*-values. Rather, we examine generalised trends in the data (i.e. consistency in the direction of the model coefficients and precision of 95% confidence intervals) to infer indications of structural biases in the data; while this is necessarily somewhat subjective, it is preferable to discarding valuable information because it did not meet an arbitrary threshold designed for hypothesis testing. We run Poisson regression models to describe the data, not to make broader population-level inferences. We therefore do not refer to *p*-values. As our interest lies in optimising the feature for retaining mothers poorly represented in the existing public health literature, in each model the reference group is the majority or dominant condition in the literature (White, graduate, and income above £35,000 (the equivalent of the median household income prior to taxes and benefits in the UK in the financial year ending 2022 [35]) and breastfeeding, while age is continuous in years.

To examine whether early postnatal experiences are linked to app feature usage, we calculated how often each participant reported any of 12 survey outcomes (e.g., others feeding the infant, negative experiences, infant feeding problems, positive infant behaviours, or receiving no support - all response variables are listed in Fig. 6) divided by the total number of surveys they completed during the study. The correlation of the 12 resulting proportion variables with total usage is used to infer variation in the frequency of feature usage dependent on whether individuals reported more neutral, more ‘negative’ (e.g. more infant feeding problems, less support) or more ‘positive’ experiences (no infant feeding problems, positive infant behaviours, sufficient sleep). This division reflects the original classification of the options during questionnaire development, reducing down the number of categories to three for each outcome (behaviour, feeding, sleeping and support).

Finally, to assess the utility of our daily prospective design we explored individual-level daily change across response items (Fig. 6). To do so, we created a composite score to capture the proportion of day-to-day changes participants made to each of their responses in the survey (e.g. day one to two, day two to three, day three to four and so forth). This score is on a scale 0 – 1, with 0 indicating responses to an item never changed (e.g. the infant was only ever breastfed) and 1 indicating a different response from each day to the next was reported (e.g. mothers received support from different people each day).

#### Qualitative analysis

Content analysis was employed to examine the interview data from the 14 women, each interviewed four times. In the preparation phase, the transcribed texts were read, combined by week and individual, and re-read to gain a sense of the ‘whole’ narrative [36, 37]. Subsequently, a systematic coding process was undertaken using an inductive approach, without predefining themes for identification. This involved line-by-line coding, creating new categories as topics emerged from the transcript where participants consistently mentioned key themes [36]. These codes were then combined into five initial major themes, again without predefinition, reflecting central concepts in the participants' narratives. This process was validated by discussions between researchers and stakeholders. During this process the fifth theme ('negative reinforcement') was combined with the theme ‘reflection’, since negative reinforcement was understood to be the negative outcome of reflection. Once the four themes were finalised, the text was re-coded with the four thematic categories and representative quotes were identified, presented here. Fictitious names are used alongside direct quotes for anonymity.

## Results

### Sample characteristics

**Fig. 1:**
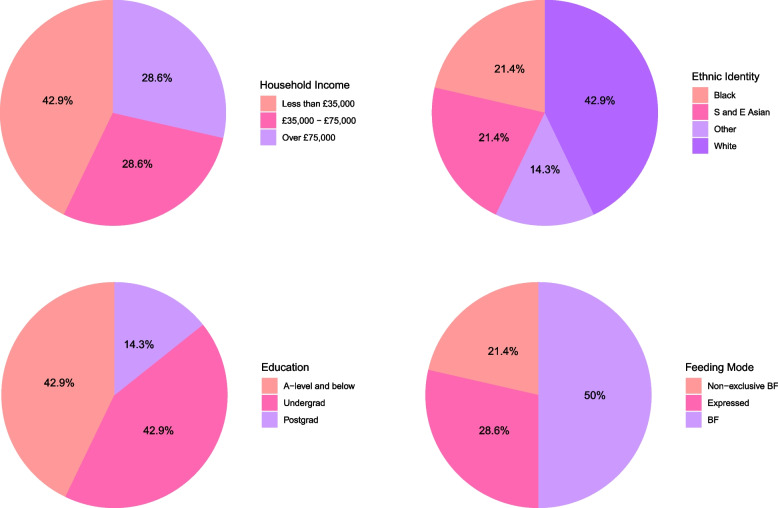
Sociodemographic features of the final sample (*n* = 14). Please note the income variable has been reduced because of small sample sizes in some categories. Abbreviations: breastfeeding (BF), South and East Asian (S and E Asian). The full breakdown can be found in Table S1

The sociodemographic features of the 14 participants are presented in Fig. 1 and Table S1. The average age of participants was 31.2 years (SD = 5.5), ranging from 19 to 38 years and all infants were aged 2 months or less. A slight majority of the participants had graduated from university (57.2%, *n* = 8), while the largest proportion earned a *household* income of less than £35,001 (42.9%). Of the 14 women, six selected ‘White’ as their ethnicity (42.9%), and the second largest ethnicity reported was ‘Black’ (21.4%, *n* = 3). It is notable that not a single person signing up for the study selected ‘only formula feeding’ as the current infant feeding status, and the majority (over 52%) reported exclusively breastfeeding. Of the selected sample, 50% of women breastfed exclusively without expressing milk, while two individuals (14.3%) reported using formula alongside breastmilk at some point since the baby’s birth.

The pilot study was run over 28 days, where women received push notifications to their phones in the morning reminding them to fill out the daily survey. On average, participants responded to the survey on 22.4 days (SD = 4.5), representing 79.7% of possible days. This value ranged from 15 days (53.6% completion) to 30 days (one participant used the daily reporting for two more days beyond the study). Overall then usage was high, however this is within the context of the small-scale pilot study where participants were incentivised to use the app. When asked to rate their overall experience of using the daily reporting, the averages were consistently high over the four weeks;. out of a maximum score of 10, the week 1–3 average was 8.5 (SD = 1.2, 1.3 and 1.4), and week 4 was 8.2 (SD = 1.7). At each interview, all women reported that the length of the survey and its daily frequency was acceptable. On average, women reported the survey taking them 2.7 min (SD = 1.3) with a minimum of 1 and maximum of 5 min. Finally, the majority of women (71.4%, *n* = 10) reported ‘yes’ when asked in the last week whether answering the same questions each day was ‘acceptable’, while 28.6% reported ‘no’. Overall, these results suggest the daily reporting was quick to complete, user experience generally was a positive experience and daily completion rates were high.

### Associations between maternal characteristics and daily usage, duration and experience

#### Daily usage – number of days participants filled out the survey

**Fig. 2:**
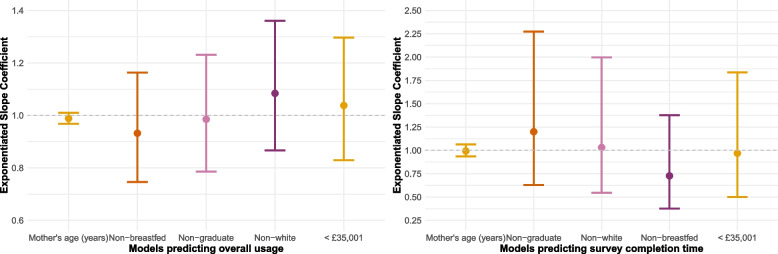
Plotted relative risk ratio from the Poisson regression models for **A**) usage and **B**) duration, *n* = 14. The dashed line at 1 represents no effect

The results from the Poisson regression models estimating daily usage are presented in Fig. 2A and Table S2. As the point estimates for both non-White and lower income participants are clearly positive, and those for lower education and maternal age very close to the null, there is no indication these characteristics are associated with less usage. However, non-exclusively breastfeeding participants have a negative point estimate suggesting reduced reporting, with significant uncertainty given the wide confidence intervals.

#### Reporting duration

The results for the analysis of time taken to complete the survey are presented in Fig. 2B and Table S3. We see no associations with income, ethnicity or age. There is a moderate positive effect for education (RR = 1.2). The model estimated value of time taken for graduates is 2.5 min, which increased to 3 min in non-graduates. Non-exclusively breastfeeding mothers tended to complete the survey quickest (model estimated = 2.29 min compared to 3.14 min).

#### User experience

**Fig. 3:**
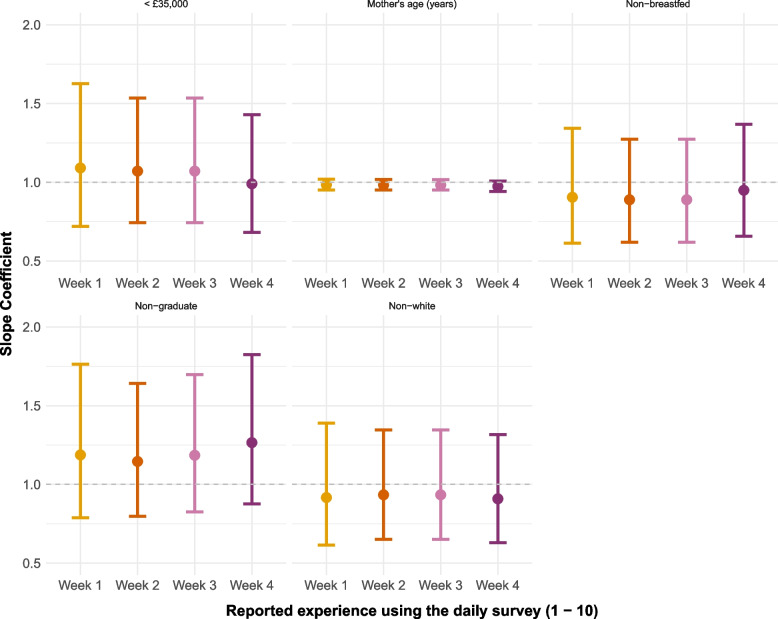
Plotted relative risk ratios from the Poisson regression models for weekly experience rating (1–10) using the daily reporting, *n* = 14. The dashed line at 1 represents no association. Results are separated by model, and the x-axis is the week of reporting

The Poisson models exploring overall user experience are presented in Fig. 3 and Table S4. Here, we are interested in two trends. Firstly, are typically underrepresented groups consistently reporting poorer experiences (represented by point estimates below the line at 1)? Secondly, is there a deterioration in experience through the weeks? Results suggest there is no association with age, and little with ethnicity and non-exclusively breastfeeding. For education, the highest ratings occurred in week 4 for non-graduates (model estimated experience rating of 9.33 in non-graduates compared to 7.38 in graduates). Meanwhile, by week 4 lower income participants no longer diverged from those of higher income (model expected experience rating for those earning £35,001 or above = 8.25 compared to 8.16 in those earning less than £35,000). This suggests that over time either non-graduates and lower income individuals experience of the feature improved, or graduates and higher income individuals’ experiences grew worse. This may be related to issues surrounding repetition which are discussed below.

#### Acceptability of repetition

**Fig. 4:**
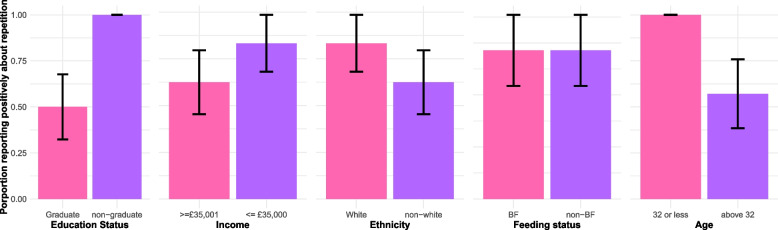
Bar charts represent the distribution of responses to whether the degree of repetition was acceptable by sociodemographic feature. Here, age has been separated into a binary variable (aged 32 or less, or above 32 years). A bar of 1 represents when everyone in this group reported the repetition acceptable, anything below one indicates some found it unacceptable. Error bars are standard error of the means. Abbreviations: breastfeeding (BF)

During the interview on the fourth week, participants were each asked if they found the level of repetition in the daily reporting acceptable or not. The proportional distribution of responses can be seen in Fig. 4. We found no difference in acceptability by feeding status, but the other categories suggest some trends. Primarily, all those that reported the repetition to be unacceptable were graduates (50% of graduates reported it acceptable, compared to 100% of non-graduates) and aged above 32 (57.1% of individuals aged above 32 reported acceptable, compared to 100% of those aged 32 years or less). Furthermore, while the proportions were more similar, those earning above £35,001 reported less acceptance (62.5% compared to 83.3% of those earning less than £35,000). Finally, we see that non-White women reported lower levels of acceptance (62.5% compared to 83.3%). Overall, while we remain cautious of strong inferences with limited data, it appears that repetition was less acceptable for older participants from wealthier, more educated, and *perhaps* non-white backgrounds.

### Associations between postnatal experience and usage

**Fig. 5:**
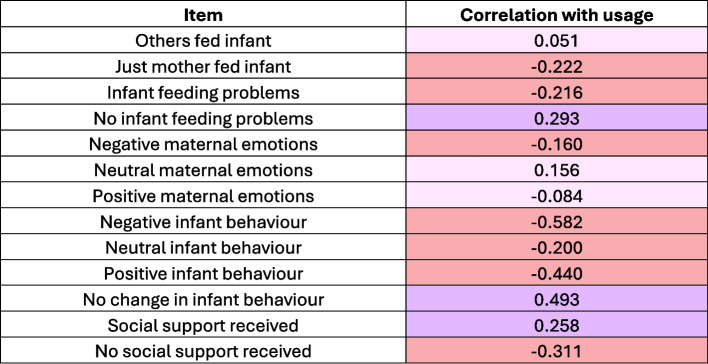
Correlation between for the relationship between the total usage and the proportion of different outcomes were reported. Purple shades represent positive correlations, pink shades negative, and pale shades small correlation coefficients. Behaviour in this context refers to questions about the infant’s behaviour, emotions to the mothers’ emotions associated with infant feeding

The correlations between participants’ postnatal experience and their daily reporting frequency range from -0.58 to 0.29 and can be seen in Fig. 5. In general, while the correlation coefficients are mainly small, poorer experience correlated with less usage as indicated by pink shading (i.e. negative correlations). For instance, the more frequently individuals reported negative experiences, i.e. negative infant behaviour, infant feeding problems, no social support, and solo-feeding the infant, the fewer days they used the feature. This association was strongest for negative infant behaviour with a correlation coefficient (r) of −0.58, followed by no social support with r = −0.31. However, individuals who more frequently reported positive infant behaviour also reported less (r = −0.44), as did those who reported positive emotions (r = −0.08) more often. Finally, we see individuals who on more occasions reported social support (r = 0.26), no infant feeding problems (r = 0.29), and no change in infants’ behaviour (0.50) used the feature on more days. This is an indication that those who have negative experiences may require further incentive to engage with the app feature regularly.

#### Daily variation in the content of maternal reports

**Fig. 6:**
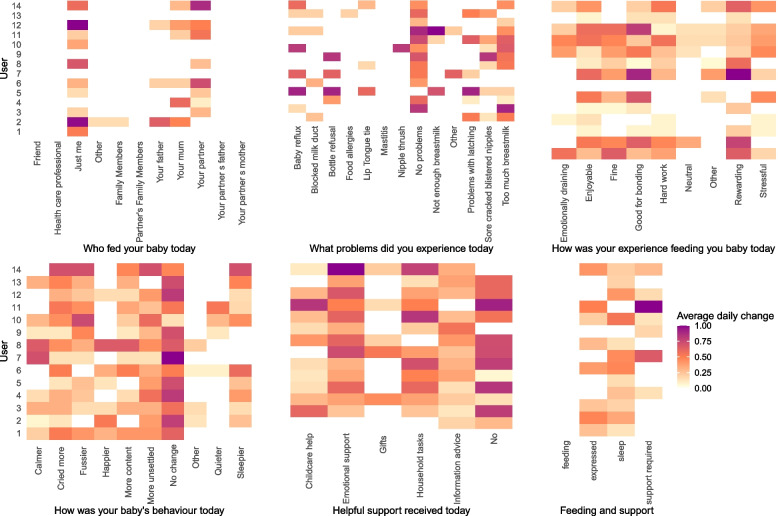
Heat map for each of the item groups (who fed the infant, infant feeding problems, mother’s experience, infant’s behaviour, helpful support and types of feeding and support received) day-to-day change. Darker shades indicate increased daily changes – a value of 1 (purple) indicates that a user’s response changed every single day for this item, while a value of 0 (white) suggests this response never changed but some response was always documented (there are no missing data points). Each user is denoted by a row on the y-axis

Participants frequently changed responses to the questions in the survey. This happened most notably in experience categories with a null condition (i.e. no problems in infant feeding problems, no change in infant behaviour and no support), as indicated by the darkest shades in Fig. 6. This suggests that participants frequently moved from a state of not experiencing problems or not having support to some type of problem or support, but which exactly, varied. We saw no variability in feeding mode (i.e. they always gave either breastmilk exclusively or mixed breastmilk with formula), however mothers moved in and out of expressing breastmilk on 25% to 50% of days. While there was lots of variation between just the mother (‘just me’ response) feeding the infant and one particular supporter (i.e. ‘your partner’ or ‘your mum’), there was little variation in who else fed the infant (most responses are white indicating no change). Infant feeding problems associated with milk supply, latching and baby reflux and bottle refusal were most changeable, suggesting the importance of collecting granular data for better understanding their impact on breastfeeding outcomes.

### Qualitative results

A content analysis of the interview transcripts produced four themes.

#### Simple, quick, easy and repetitive

All apart from three participants directly mentioned that they felt a likeable feature of the daily reporting was that it was easy to do. Participants reported that the daily reporting was very quick to do, making it low investment, particularly when they are busy which helped them maintain their motivation. For example, Chloe said: “Yeah, I feel fine as because it's not like a longwinded process…the questions are simple and quick, you know?” This process was supported by the feature being ‘easy’, as stated by Alex (week 2) “It's easy to read. Something that I like is that simple. There's no, like, you know, animations or anything…”.

Participants mentioned that the questions being the same daily meant they felt confident in answering them and they got quicker over time. This highlights the importance of repetition and consistency. Roxy (week 4) reported that “It just kind of got quicker as it went on really, because I knew what the questions were.” Charlotte, who originally reported the daily survey taking 1 – 2 min reported a significant shorting of time by week 4, supporting her engagement “It didn't take any time at all because I knew what was coming… it was quite easy to do”. When participants were asked if they would be happy to continue reporting over the next 10 weeks, 10 of the 14 (71.4%) reported they would be happy without further functionality being added. The most commonly cited reason for this was that it was so low investment (7/10).

#### Habit formation and routine

Related to the repetitive element to the daily questions was participants’ recognition that it was easy to remember completing the daily reporting because they had formed a habit; using the app feature became part of their daily routine. Out of the 14 participants, eight reported that filling out the questions had become a habit for them, so they automatically remembered to do it (rather than relying on the push notification). This habit formation helped maintain their motivation as they did not think about *having* to do the survey, they just did it. For instance, Roxy in week four reported “It's just kind of become a habit now. I don't really notice it; I just do it”. There was also the recognition as a result that daily reporting helps build this routine because it was so frequent. Charlotte (Week 3) reported “Doing it daily, it's not a problem. You got into a routine. Otherwise, I think you'd lose that and then you'd forget to do it if you're doing it sporadically”.

However, the majority (11 of the 14) of participants felt that they would have struggled or found it complicated to fill out the daily survey straight after childbirth and during the first 14 days postpartum, given the intensity of the period and the lack of routine in their daily lives at this time. For instance, Fiona reported (week 3) “[for the first few weeks] my routine was totally out of kilter. There were times I didn't even look at my phone”. This dynamic is particularly emphasised by those who experienced difficult childbirths with limited social support. For instance, Alex in week 2 reported: “Personally, I had no help… So, you know, it was a lot, especially in the beginning because I had a C-section and it was a bit difficult to recover from it… [I] probably [could have started completing the questions] after the first month just coming out of that, you know, newborn fog month, roughly”. There was a recognition, however, that because midwives and health visitors are asking mothers to accurately report their infants’ feeding and nappy changes there is a need to ‘track’ infant feeding somewhere, which could mean the habit for completing the survey is developed earlier on in the postnatal period despite the intensity of the period. This was stated by Charlotte (week 3): “But if there was a simple way of like tapping a button or to do with like wet nappy or whatever… if you could note it in an app and that would be really useful”.

Nonetheless, three women did report feeling they would have benefited from completing the daily questions from early in the postnatal period, because a lot was changing, and they needed more support during this period. For these individuals, filling out the survey was more useful when more was changing on a daily basis.. For instance, Chidinma (week 3) reported: “…I think probably right after [the birth] would have been more, would be more helpful. For first time moms like me”. Likewise, Charlotte reported (week 3): “Yeah, I think it'd be useful and it's not mandatory. So, I mean if you don't have time for it that day, it's not the end of the world. But I think when you do get time and once you get used to the questions, it's so quick…”.

#### Reflection, acknowledgement and space

A third key theme that emerged is 'Reflection, acknowledgement and space.' This theme encapsulates the idea that, despite the absence of any feedback, participants found value in the opportunity to pause and reflect. Several participants likened this to documenting their experiences in a diary, e.g. Fiona (week 1): “… it's almost like a little diary…” or discussing things with a group of friends or other mothers, e.g. Adeline (week 1): “I feel a little bit more like it will just made a bit easier because it's like you're talking to someone, you're telling someone how you're feeling”. As a result, participants frequently (8 of the 14 participants) discussed looking forward to and enjoying the process of daily reporting. For instance, Chloe reported (week 3): “Really, I haven't lost any motivation and I definitely think each day like, *ohh*, I got to fill in the survey, yeah, So I find it quite enjoyable to be honest”. Charlotte (week 4) in particular mentioned the sense of stepping back and reflecting: “I was changing my answer depending on whether my daughter was, you know, fussier or the same or slept more. So that question particularly was helpful for me to reflect back and think actually has anything changed? And then it made me think, have I changed anything to make that change happen?”.

Reporting for some participants also provide a sense of progress, monitoring their actions and what they saw as consequences of actions. This was particularly salient for those who were breastfeeding while facing significant infant feeding problems. For instance, Fiona (Week 1) reported: “I don't know maybe it's like a little endorphin type thingy. When I press that I've breastfed again, and I'm not bottle feeding and you know I feel all right now when it asks you like how do you feel? And also like with my first son, I stopped breastfeeding at the beginning because of like mastitis and stuff like that. And even though I've had it this time, I've continued to breastfeed.” Megan (week 4) reported similarly that “But to be able to have that moment, to see that kind of progress, oh, it's a bit of a mastery as well… I think, a bit of gratitude for myself”.

This space and reflection prompted participants to step back, consider their daily experience and if they felt there was an issue, they would seek further support or discuss how they were feeling with their partner or another important person in their life. This, the participants reported was important because it helped bring on a change in their behaviour. For instance, Jolie (Week 4) reported “I think it's reflecting on if you kind of had a string of not so great responses and where the questions around the prompts of ‘did you get any support on this’, ‘Did you go to any classes’ sort of in a way prompted me to act and do something about that and seek out some baby classes”.

However, some participants felt that if they did repeatedly report negative responses this could amplify this negativity. For instance, one participant [Scarlett, week 4] said that having to report the infant feeding problem of ‘bottle refusal’ for her daughter on a daily basis was becoming more and more frustrating: “It's been like nearly two weeks now and she's not taking the bottle and I think it can because it's almost like over focusing on that and that becomes the big thing…”. Likewise, Isabella (Week 4) reported that having to record she was feeling anxious a lot made her feel more anxious about this – reinforcing the negativity: “Oh well, I keep saying that I'm quite anxious and … maybe that would make me feel a little bit worse.” However, she also stated that it is important to recognise a problem to fix it: “But I know that I need to overcome that by … recognising what I am anxious about…”.

This theme of documenting negative responses as empowering individuals to seek help or change was repeated by multiple participants. For instance, Violet (week 4) stated that after documenting that her baby was fussier or crying more than usual then she would look at another app to find if there was a developmental reason. Doing so helped her deal with the ‘stress and upset’. Similarly, Ophelia (week 4) discussing with her partner when she was reporting lots of negative responses for her daughter and she wanted to know what was happening: “[What made me feel better was] talking to my partner. You have to monitor this [baby’s behaviour] and talk to my partner. I would be like OK, I realised that actually she's a lot more fussy than she is calm, because I stay at home a lot and I'm always home. And when I do actually go out to see family that, you know, that's when she's fussy.” Participants suggested the trade-off between amplification and empowerment of documenting negativity may be altered by providing further information or support from within the feature; for example, Charlotte suggested: “Because there's no, like, feedback from the app feature in terms of like, I don't know, a graph or whatever. I guess it can feel quite negative. But then if there was feedback, then you would see like, like a trend and maybe that would be helpful.”

Therefore, participants enjoyed recording their daily experiences in the survey because of the chance to reflect, acknowledge and check-in with themselves and their progress. Participants acknowledged this may magnify negative feelings, but this was a trade-off necessary to seek out further help or support for these negative responses.

#### Repetition is difficult and annoying

The final theme from the content analysis was that some participants, after four weeks, felt the repetition in the daily questions to be annoying, frustrating and pointless. This theme ties in with the quantitative results which highlighted that certain participants reported less acceptance of the repetitive nature of the questions.

Participants largely reported that while the process of filling out the daily questions was ‘fine at first’, they became bored with the process. While other participants liked the fact that the questions remained the same each day, this group found the lack of variation tedious. For instance, Fiona reported (week 4): “It was fine at first, and then literally this last week I've been thinking that it's quite yeah, I guess the app is quite boring and then the questions are very repetitive”. Likewise, Ekeanuamu reported (Week 4) that “it’s become like a bit boring because I mean normally select the same question like every day.” Participants mentioned that regardless of how quick or easy the survey was to do, their motivation was waning because of the lack of variety. Another participant observed that earlier in the process of using the survey their baby was also younger, and more was changing overall. So perhaps it is not that the questions are the same, but the participants responses stopped varying so much by week 4. Fiona commented (week 4): “They were often the same, particularly in terms of how you feed your baby. You know, the support that you get, how much sleep you get. You know, those things did not change much” in contrast to the earlier postnatal when she was ‘finding her feet again’ and had more interest in answering the questions.

In particular, participants linked the issue with the repetitive nature of the questions to a lack of feedback. Megan (week 2) reported “It's more the so what again? Because then the answer I guess for me it's like, what's the point of doing it if I know that most of the time my breastfeeding is enjoyable, for example…It's like, but I'm not benefiting anything from it. It's a bit tedious do it.…So yeah, I did find it repetitive, but if it's useful then I wouldn't”. Participants made a number of suggestions for the form this feedback could take. Fiona talked about a fitness app she uses to record calories and activities every day because “then at the end of the week I really looked forward to the summary that you get” via a dashboard on the app; others suggested email roundups. A further suggestion was being signposted to services based on responses; for instance, Baby Buddy has a video library of information and tips for common infant feeding problems and individuals could be provided with a relevant link if reporting a particular issue. Therefore, while boredom and attrition in usage are potentially drawbacks, they may be overcome with the inclusion of more feedback and benefits for participants.

## Discussion

We conducted this study to explore the feasibility of a methodological tool which 1) collects dense data 2) daily, 3) during the hectic early postnatal period, and 4) does not systematically exclude less privileged women by 5) adding value to participants. Participant attrition is a major concern in any study which requires multiple follow-up points [38]. It reduces sample sizes, wastes effort and results in non-random missing data when there are sociodemographic predictors of attrition [39, 40], which may prohibit causal inference [41]. These concerns are magnified when the data collection occurs at a high frequency over a prolonged period when participants are particularly time burdened. However, this is precisely what is required for quantitative research into infant feeding and social support.

By following an iterative, human centred design approach to mHealth we worked with our participants in a collaborative process [31, 32] to develop the first version of a survey ‘feature’ within the existing Baby Buddy app. The results presented here overwhelmingly demonstrate that this initial version was successful in meeting our aims. Firstly, even in the minimum viable product stage, women found completing the survey ‘easy’ because it became a habit *and* they enjoyed doing so because it gave them time and space to reflect. This is supported by participants using the app feature on the majority of days, spending only a few minutes reporting and rating their user experience highly. Secondly, we followed a non-random sampling strategy to ensure our participants represented a diverse range of women commonly under represented from similar studies. Our quantitative analysis found no evidence that sociodemographic features were associated with women’s usage, enjoyment and time spent reporting, although the sample size was rather small to draw firm conclusions. While past researchers have been concerned that participants will not engage with long repetitive surveys repeated over several weeks or months, or quickly drop out of studies, the evidence we present here suggests this is not necessarily the case.

Our approach imposed as little structure as possible, allowing users’ needs and motivations to guide development and using a ‘less is more’ approach [31, 42]. At its core, this approach is empathic and collaborative. This was well suited to our requirements that the survey was regularly used by participants for a prolonged length of time. Such commitment requires significant buy-in, suggesting users must gain something in return. As a result, our slow-iterative and agile feedback process ensured participants’ voices were incorporated for the duration of the study and will now be fed into version 2 of the feature. This is particularly important for voices which are less well represented in research or policy [43]. While listening and being responsive to users’ requirements from the very start extends the length of the development process, it maximises the relevance of the end product [31]. Such an approach helped us both make small iterative changes during the pilot study, but also created a list of additional elements for development in the next version. This underscores the value of co-produced research, where participants gain the most value, and the research better reflects their reality, when they are involved from the start. For this reason, there has been increasing recognition and emphasis of co-produced frameworks within the social sciences [44, 45] which, we hope, continues to grow.

Such approaches are necessary given the complexity of interface between infant feeding, maternal mental health and social support. Across multiple of studies, it is apparent that whether breastfeeding (exclusive or otherwise) is extended by support from partners, family, friends, peer-supporters and health care professionals depends on context. For instance, various pieces of research have pointed to the varying implications of the *types* of support that supporters offer [9, 16, 46–48], how much in *need* the individual is of that support [9, 15], the perceived *accuracy* of supporters knowledge and information about infant feeding [5, 49, 50], and if advice received is *conflictual*, particularly between health care professionals [14, 51]. Furthermore, there may be dialectal tension between social norms and expectations about infant feeding and motherhood and individuals experience [5, 51, 52]. In short, early postnatal experience is complex and a careful teasing apart of these pathways is necessary to better support mothers [5], requiring approaches such as those piloted here. This is further reinforced by our results underlining how much individuals’ responses changed on a daily basis.

Despite participants receiving no built-in feedback from the feature, many reported that they looked forward to completing the surveys because of the time it gave them to acknowledge and reflect on their experiences. Such findings are in line with the therapeutic literature on journaling and expressive writing. Researchers have consistently found that participants report improvements in physical and mental health with short periods devoted to writing about their experiences [53–56]. While selecting multiple choice options on a mobile app is a long way from expressive writing, journaling may take many forms and for some can be as simple as tracking experiences on a score card [57]. Mechanistically then, what is important is that the act provides the participant with a tool for reflection, reinforcement of positive experiences, as well as acceptance of negative experiences [55, 58, 59]. By helping participants with cognitive processing, organising their experiences and perhaps accepting negative emotions, users received a benefit from the daily documentation of their experiences without having any actual feedback [54, 55].

Nevertheless, a concern remains that ‘reflection’ reinforces negative experiences, presenting an ethical concern. This element in particular will be the focus in future development research, and we will take steps to address these concerns. For instance, we will explore these questions in-depth in focus groups with more users. Furthermore, we will include additional features in the next phase which will – we hope — mitigate negativity. For instance, participants will be able to skip questions, and automatic signposting is being built into the feature, so participants who flag a particular issue will be provided links to informational resources at the end of the survey. A short term increase of negative feeling has been observed in the journalling literature, but this is typically surpassed with long-term improvements across multiple domains [54]. Our participants also reported that it is important to recognise and accept how you are feeling to be able to take steps to address it and improve. Therefore, the risks associated with reflection may be minimal over the long-term; large-scale, longitudinal tracking in the next stage of development will ascertain whether this is the case.

Our participants also discussed how easily reporting on a daily basis became a habit for them. We specifically probed if reporting daily was ‘acceptable’ to our participants, and while a few participants reflected that the repetition became difficult, many recognised that reporting on the daily basis actually helped them remember. This is in line with the psychology literature which highlights that a habit is learned through repeated action, which forms context-response associations in memory so habits become easier to do despite changes in goals, outcomes, motivation or barriers [60]. This reflects what our participants reported in interviews, and why completing a simple daily survey can be built into individuals routines, even when their lives are hectic. The subgroup of participants who did not find repetition acceptable had higher educational attainment and income, and their boredom likely underpins the less positive user experience ratings in these demographics. Our qualitative analysis revealed that those who became ‘bored’ by the repetition desired feedback on their input. Thus, they suggested that a daily or weekly summary of their experiences, in a dashboard or email format would help; this will now be included in further feature development. Of course, these findings do not fully apply to ‘real-life’ since in this intensive study women received a small payment to thank them for their considerable input and time. Therefore, the focus of the next stage of piloting is to uncover if the non-financial incentives within the feature (signposting, reflective acknowledgment, weekly summaries of recorded experiences) are sufficient to motive regular use over a long time period.

Our participants began using the feature some weeks after birth; however, our ultimate aim is to collect data starting immediately from birth which might be particularly challenging when individuals’ routines are extremely disjointed. However, feedback from participants indicated that additional tools within the feature would promote their early engagement, such as the ability to track events which are required by midwives. It is noted in the mHealth literature that users often wish to be able to set goals and record information in as barrier-free a method as possible [42]. This will be developed for the next stage of the feature development.

Our second core aim was to ensure that key sociodemographic metrics (educational attainment, ethnicity and income) were not associated with our participants usage, time spent reporting or experience of the feature over the four weeks. While our sample size was small, across the point estimates and the confidence intervals we saw no indication of bias against women who are more frequently underrepresented in the quantitative infant feeding literature. One trend which did arise from our analysis *may* suggest a trend that women who were not exclusively breastfeeding spent less time filling out the survey, reported lower experience ratings and completed the survey on fewer days. Since we had no women sign-up who were *only* formula feeding their infant it is hard to unpick these results and this will be a focus in future research. From the qualitative analysis, it may be the case that women who reported breastfeeding in the face of complications may have felt a greater sense of reward or ‘buzz’, thereby experiencing more positive effects from journaling and easier reporting habit formation.

Finally, in our quantitative results, we saw a tendency for individuals who received less support and had more negative postnatal experiences to engage with the app feature less. It appeared when you have little support from family members, or others to help in the home, you cannot devote energy to additional tasks regardless of how ‘easy’ and ‘quick’ they are. Again, this will be an aspect explored with larger samples in the next stage of research. It is important to note that we did not consider all aspects of exclusion in this study, specifically ability to read English, and women who have learning or intellectual disabilities. Such women are commonly excluded from research on reproductive and sexual health, and require inclusion in the development of material and methodologies to ensure equality in access [43]. We kept the text as simple and easy to read as possible, making it accessible to the average British adult. Nonetheless, further improving this will be a consideration in future versions. While infographics are a common solution for minimising the use of text in apps, this is also may also introduce barriers; for instance, women with intellectual disabilities may misunderstand visual metaphors included in infant feeding resources, and report a preference for simple and non-abstracted images [43]. While the current version of the feature does not contain images, this will be taken into future design consideration. In the next stage of development, we will work to purposely sample women who do not speak English as a first language and have learning or intellectual disabilities.

## Conclusion

Our pilot study set out to explore the feasibility of a methodological tool to collect dense, prospective daily data on infant feeding and social support from a diverse range of postnatal mothers in the UK. Overall, the first version of an mHealth data collection feature, embedded within the widely used Baby Buddy app, achieved our aims by being easy and quick to use, motivating consistent daily use by acting as a tool for reflection and quickly becoming a habit. Following an iterative, co-produced framework we gained extensive insights from participants for the next steps to develop the feature further, specifically to include feedback and tracking mechanisms. Our results underscore that it is possible to develop methodological solutions to common problems in prospective data collection, particularly for infant feeding in the hectic postnatal period. Our results also speak to the importance of inclusive, collaborative and empathic methodological development, as embodied by human-centred design. As a result, our findings are relevant to many in the social sciences and public health and indicate a clear path forward for infant feeding studies which require new approaches to unpick the complexity of the postnatal experience.

## Supplementary Information

Supplementary Material 1

## Data Availability

The quantitative dataset supporting the conclusions of this article is available in the OSF project https://osf.io/yqsnd/ [10.17605/OSF.IO/YQSND].
