# Supplementary material for: Collecting real-time infant feeding and support experience: co-participatory pilot study of mobile health methodology
Source: Int Breastfeed J. 2025 Apr 3;20:23. doi: 10.1186/s13006-025-00707-7 (PMC11969986; doi:10.1186/s13006-025-00707-7)
Supplement: Supplementary file 1 — Supplementary Material 1 [file 13006_2025_707_MOESM1_ESM.docx]

**Collecting real-time infant feeding and support experience: co-participatory development of mobile health methodology**

Abigail E. Page^1,2^, Emily H. Emmott^3^, Rebecca Sear^1,2^, Nilushka Perera^4^, Matthew Black^4,5^, Jack Elgood-Field^4^, Sarah Myers^6^

^1^ Centre for Culture and Evolution, Brunel University London, UK

^2^ London School of Hygiene and Tropical Medicine, Population Health, UK

^3^ University College London, Department of Anthropology, UK.

^4^ Best Beginnings, London, UK

^5^ Department of Health and Social Care, London, UK

^6^ Max Planck Institute for Evolutionary Anthropology, Leipzig, Germany

**Keywords:** human-centred design; infant feeding; social support; mHealth; co-production

**Result tables**

**Table S1:** Sample summary statistics

|  | **Count** | **%** |
| --- | --- | --- |
| **Education** | | |
| A-level and below | 6 | 42.86 |
| Undergrad | 6 | 42.86 |
| Postgrad | 2 | 14.29 |
| **Income** | | |
| Less than £35,000 | 6 | 42.86 |
| £35,000 - £75,000 | 4 | 28.57 |
| Over £75,000 | 4 | 28.57 |
| **Ethnicity** | | |
| Black | 3 | 21.43 |
| Other | 2 | 14.29 |
| S and E Asian | 3 | 21.43 |
| White | 6 | 42.86 |
| **Feeding status** | | |
| BF | 7 | 50.00 |
| Expressed | 4 | 28.57 |
| non-exclusive BF | 3 | 21.43 |

**Table S2:** Results from the Poisson regression models for usage.

|  | **Slope Estimate** | **Lower CI** | **Upper CI** |
| --- | --- | --- | --- |
| **Ethnicity** | 1.084 | 0.866 | 1.360 |
| **Income** | 1.038 | 0.829 | 1.297 |
| **Education** | 0.985 | 0.786 | 1.231 |
| **Feeding** | 0.932 | 0.746 | 1.163 |
| **Age** | 0.988 | 0.968 | 1.010 |

**Table S3:** Results from the Poisson regression models for time take to complete the survey.

|  | **Slope Estimate** | **Lower CI** | **Upper CI** |
| --- | --- | --- | --- |
| **Age** | 0.993 | 0.935 | 1.063 |
| **Education** | 1.200 | 0.629 | 2.273 |
| **Income** | 0.970 | 0.501 | 1.836 |
| **Ethnicity** | 1.031 | 0.545 | 1.997 |
| **Feeding status** | 0.727 | 0.376 | 1.377 |

**Table S4:** Results from the Poisson regression models for weekly experience rating (1-10).

| **Model** | **Week** | **Slope Estimate** | **Lower CI** | **Upper CI** |
| --- | --- | --- | --- | --- |
| Education | 1 | 1.188 | 0.788 | 1.764 |
| Income | 1 | 1.091 | 0.720 | 1.626 |
| Ethnicity | 1 | 0.917 | 0.615 | 1.389 |
| Feeding | 1 | 0.905 | 0.613 | 1.343 |
| Age | 1 | 0.984 | 0.951 | 1.019 |
| Education | 2 | 1.146 | 0.797 | 1.642 |
| Income | 2 | 1.071 | 0.743 | 1.535 |
| Ethnicity | 2 | 0.934 | 0.652 | 1.345 |
| Feeding | 2 | 0.889 | 0.619 | 1.274 |
| Age | 2 | 0.983 | 0.951 | 1.018 |
| Education | 3 | 1.185 | 0.825 | 1.698 |
| Income | 3 | 1.071 | 0.743 | 1.535 |
| Ethnicity | 3 | 0.934 | 0.652 | 1.345 |
| Feeding | 3 | 0.889 | 0.619 | 1.274 |
| Age | 3 | 0.982 | 0.950 | 1.017 |
| Education | 4 | 1.266 | 0.877 | 1.825 |
| Income | 4 | 0.990 | 0.681 | 1.429 |
| Ethnicity | 4 | 0.909 | 0.630 | 1.316 |
| Feeding | 4 | 0.949 | 0.657 | 1.369 |
| Age | 4 | 0.974 | 0.942 | 1.008 |
